# Synthesis and Enhanced Electrocatalytic Activity of Platinum and Palladium-Based Nanoflowers Supported on Reduced Graphene Oxide

**DOI:** 10.3390/molecules29225462

**Published:** 2024-11-20

**Authors:** Jiefa Shen, Ruigang Xie, Sai Zhang, Daixiang Chen, Shenghu Yan, Lingli Zhou, Jiayin Wang

**Affiliations:** 1 School of Pharmacy & School of Biological and Food Engineering, Changzhou University, Changzhou 213164, Chinaysh@cczu.edu.cn (S.Y.); 2 School of Environmental Science and Engineering, Changzhou University, Changzhou 213164, China

**Keywords:** graphene oxide, palladium/palladium-based nanoflower, electrochemical oxidation reaction, hexadecyltrimethylammonium bromide

## Abstract

Graphene-supported flower-shaped platinum and palladium nanomaterials assisted by hexadecyltrimethylammonium bromide have been successfully developed. Compared with the catalyst reduced by the redox reaction between metal precursors and graphene oxide, the flower-shaped catalyst obtained using reduction in active hydrogen atoms exhibits enhanced catalytic activity in alcohol electrocatalytic oxidation reactions. Repeated cyclic voltammetry and chronoamperometry tests both indicate that the prepared catalyst has excellent stability.

## 1. Introduction

In recent years, environmental pollution has led to an increasing demand for clean energy, and fuel cells have gradually gained popularity among people [1,2,3]. Catalytic materials have always been key to fuel cells. Sustained and stable electrochemical performance generally requires high requirements compared to surface area, active sites, morphology, and size [4,5,6]. To meet these requirements, more nanostructures have been designed, including zero-dimensional materials in the form of quantum dots [7,8,9] and nanoparticles [10,11,12], one-dimensional materials in the form of nanowires [13,14,15] and rods [16,17,18], two-dimensional materials in the form of layer materials [19,20,21], and three-dimensional materials with spatial divergence [22,23,24,25]. The network nanoflowers structure with more linear connections inside has attracted the attention of researchers due to its active sites with more reactant contacts [26,27,28].

Nanomaterials with specific morphologies usually require surfactants and small molecule adsorbents (such as halide ions [14,29,30,31], amine molecules [22], and carbon monoxide molecules [32]) to adsorb specific crystal planes in the crystal structure during the preparation process. From the perspective of molecular structural formula, hexadecyltrimethylammonium bromide (CTAB) can adsorb onto the surface of nanocrystals as a cationic quaternary ammonium salt with a positive charge at one end [33]. At the initial stage of the reaction, CTAB has already formed complexes with metal ions [34] and then adsorbs onto the corresponding crystal planes during nucleation and growth stages to stabilize the nanomaterials. Therefore, CTAB can play a stabilizing agent and morphology-controlling role in the synthesis process of nanomaterials [35,36]. When the reduction temperature is slightly higher or a strong reducing agent [37] is selected, CTAB-stabilized platinum or palladium is more prone to overgrowth, generating flower-shaped (or dendritic) nanomaterials.

The carrier not only disperses the catalyst to avoid agglomeration but also reduces the use of precious metals and facilitates the recovery of precious metals. Graphene is one of the commonly used carriers in electrochemistry. After oxidation treatment, the surface of oxidized graphene has abundant oxygen-containing groups, which can be used for functional modification or anchoring catalysts. Graphene oxide is used to adsorb CTAB from wastewater through surface complexation, hydrogen bonding, and electrostatic attraction [38]. Graphene oxide with a two-dimensional planar structure exhibits excellent conductivity after self-catalytic reduction or reduction by a reducing agent. The reduced platinum or palladium nanoparticles are embedded between the graphite layers, providing more space for reactants to adsorb and desorb from the surface of this three-dimensional sandwich structure catalyst.

In 1964, hydrogen spillover was first observed when WO_3_ was in contact with platinum (Pt) catalyst [39]. The hydrogen adsorbed on the catalyst surface was decomposed into active hydrogen atoms, which can freely diffuse on the support and catalyst surfaces. Active hydrogen atoms have been used to explain the reduction in graphene oxide supports [40], and there is an urgent need for active hydrogen atoms to accelerate the reduction process and simultaneously prepare supported flower-shaped catalysts. Based on the various considerations mentioned above, CTAB was selected as the stabilizer in this article, graphene oxide was used as the carrier, and clean hydrogen gas was introduced as the reducing agent to prepare graphene-loaded flower-shaped nanocomposites. As a comparison, platinum or palladium-based catalysts obtained by using graphene oxide as a reducing agent have also been prepared. All obtained reduced graphene oxide-supported platinum palladium-based catalyst was used for ethanol electrochemical oxidation reaction in an alkaline environment and methanol electrochemical oxidation reaction in an acidic medium.

## 2. Results and Discussion

### 2.1. Characterization of Palladium and Platinum-Based Catalysts Supported on Reduced Graphene Oxide

**Figure 1:**
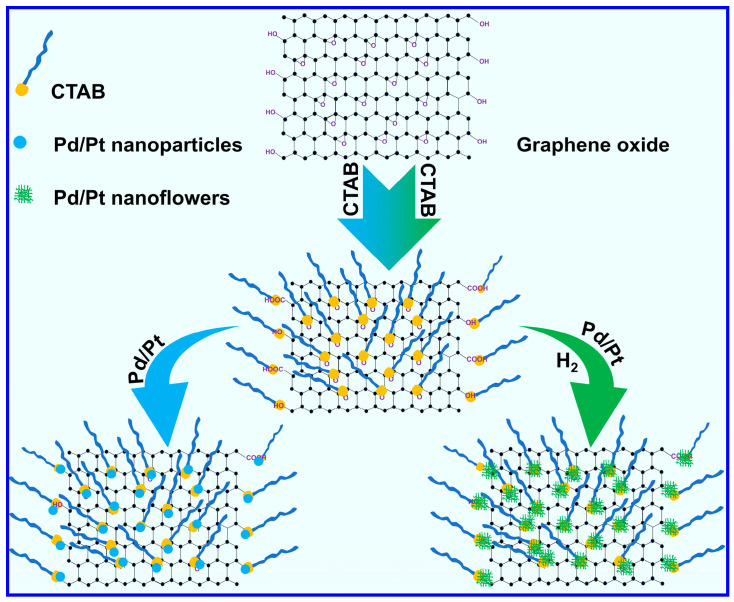
Schematic illustration of the synthesis of Pd/Pt@GO and Pd/Pt@GO (with H_2_).

The design inspiration for palladium and platinum-based nanostructures loaded with reduced graphene oxide is shown in Figure 1. CTAB adsorbs onto the surface of graphene oxide through surface complexation, hydrogen bonding, and electrostatic attraction [38]. When platinum or palladium species are adsorbed onto the surface of graphene oxide, after the introduction of strong reducing agent hydrogen gas, precious metals are loaded on the graphene surface in the form of nanoflowers, while without hydrogen gas, only nanoparticles were loaded on the graphene surface [34].

**Figure 2:**
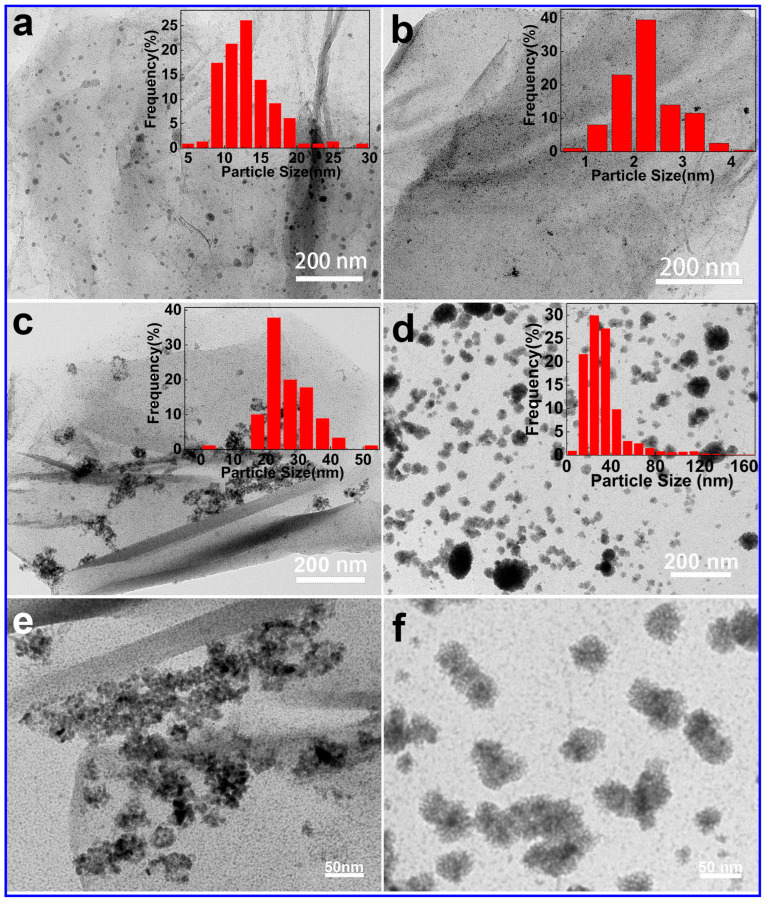
TEM image of (**a**) Pd@RGO, (**b**,**e**) Pd@RGO (with H_2_), (**c**) Pt@RGO, and (**d**,**f**) Pt@RGO (with H_2_).

Transmission electron microscopy (TEM) was used to investigate the microstructure of platinum-based and palladium-based catalysts supported on reduced graphene oxide prepared. Without the introduction of hydrogen gas, palladium and platinum nanoparticles are uniformly distributed on the surface of reduced graphene oxide, with particle sizes calculated to be 13.15 nm and 2.25 nm, respectively (Figure 2a,b). With the introduction of hydrogen, flower-like palladium, and platinum-based nanocatalysts were scattered on the surface of reduced graphene oxide. The particle size of these flower-like mesoporous catalysts was calculated to be approximately 30 nm through statistical analysis, which is 26.85 nm for palladium-based catalysts and 33.09 nm for platinum-based catalysts (Figure 2c,d). At the same time, these nanoflowers are made up of finer nanowires or rods that are cross connected (Figure 2e,f).

**Figure 3:**
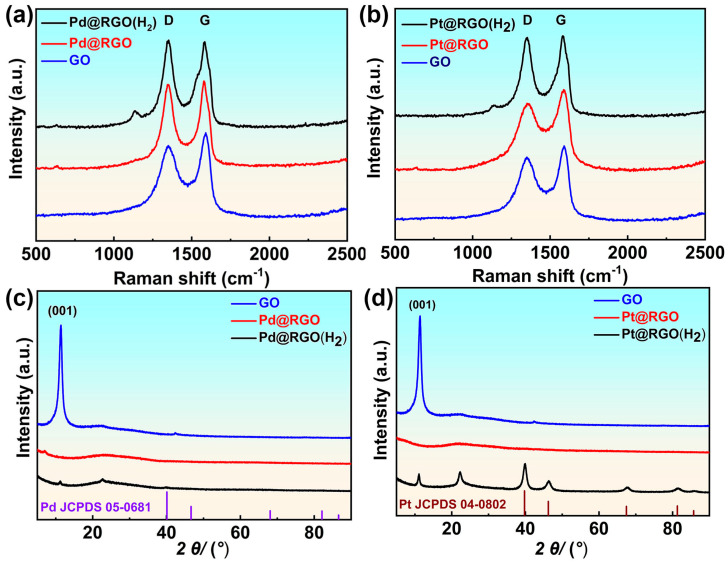
Raman spectrum and XRD diffractogram of palladium nanoparticles supported on reduced graphene oxide (**a**,**c**), Platinum nanoparticles supported on reduced graphene oxide (**b**,**d**).

In the Raman spectrum of Figure 3a,b, the two peaks located near 1350 cm^−1^ and 1580 cm^−1^ correspond to the D and G bands of graphene oxide [41,42,43]. The intensity ratio of the two peaks is commonly used to evaluate the degree of reduction and defect density of graphene oxide. In this experiment, the ratio (ID/IG) of the D-band to the G-band of the graphene oxide used was calculated to be 0.88. The ratio of palladium-based catalysts with and without hydrogen gas was 1.02 and 0.96, respectively. However, the ratio of platinum-based catalysts was 0.98 and 0.86 [44,45,46]. This suggests that hydrogen may have participated in the reduction in both precious metals and graphene oxide simultaneously. The XRD patterns of GO, Pd@RGO, and Pd@RGO (with H_2_) are depicted in Figure 3c. The XRD patterns of GO, Pt@RGO, and Pt@RGO (with H_2_) are depicted in Figure 3d. As shown in Figure 3c,d, the characteristic peak at 2 θ value 11.2° can be attributed to the diffraction of the GO (001) basal plane [35]. When hydrogen is not introduced into the reaction system, the GO (001) peak was not found in the synthesized Pd@RGO and Pt@RGO, indicating that graphene oxide had been completely reduced. A series of diffraction peaks appearing at 46.32°, 67.67°, 81.43°, and 85.75° are attributed to the (200), (220), (311), and (222) crystal planes of Pt nanoflowers [47,48,49]. The peak at 40.14° corresponds to the (111) crystal plane of Pd nanoflowers that appeared in the XRD spectrum of Figure 3c [50,51,52]. At the same time, the weak (001) crystal peak of graphene oxide is still visible in Figure 3c,d, indicating that graphene oxide has not been completely reduced and may still contain a small amount of oxygen-containing groups. The reappearance of the diffraction peak (002) at 2θ = 22.7° for Pd@RGO (with H_2_) and 2θ = 22.3° Pt@RGO (with H_2_) can be attributed to the ordered crystal structure in RGO flakes with different degrees of reduction, as well as the uneven interlayer space throughout the RGO sample [53,54,55]. After most of the oxygen-containing functional groups on the surface of oxidized graphene were reduced, the conductivity of the graphene support was significantly improved. The palladium or platinum-based catalysts supported on reduced oxidized graphene prepared may exhibit enhanced electrocatalytic oxidation activity in subsequent tests.

### 2.2. Electrochemical Performance of Prepared Platinum and Palladium-Based Catalyst

**Figure 4:**
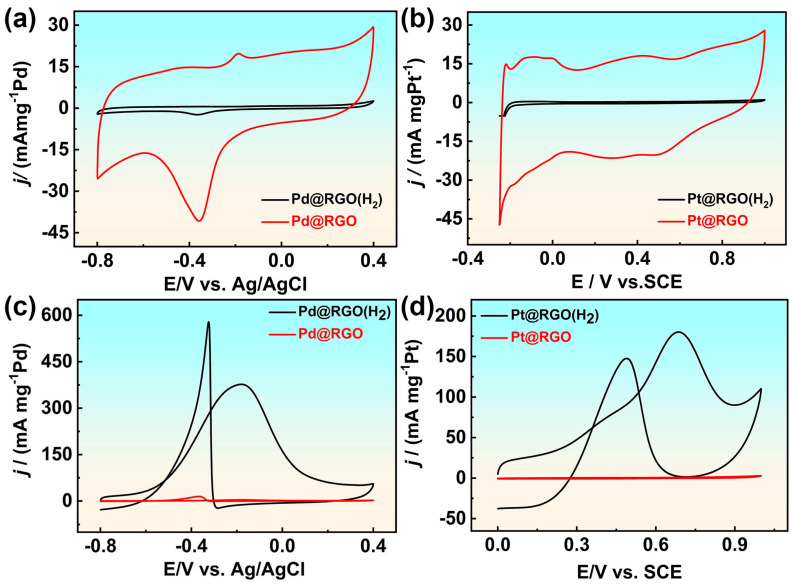
CV curves of (**a**) Pd@RGO and Pd@RGO (with H_2_) in 0.5 M NaOH solution (**c**) Pd@RGO and Pd@RGO (with H_2_) in 0.5 M NaOH with 1 M ethanol solution; CVs curves of (**b**) Pt@RGO and Pt@RGO (with H_2_) in 0.5 M H_2_SO_4_ solution, (**d**) Pt@RGO and Pt@RGO (with H_2_) in 0.5 M H_2_SO_4_ with 0.5 M methanol solution.

The electrocatalytic reaction of alcohols was used to evaluate the catalytic performance of the prepared platinum and palladium-based supported catalysts. In alkaline environments, the electrochemical area of palladium catalysts prepared by hydrogen reduction is much larger than that of catalysts obtained without hydrogen gas injection (Figure 4a). The electrocatalytic oxidation of ethanol under alkaline conditions was validated in the experiment. The electrocatalytic oxidation activity of catalyst Pd@RGO (with H_2_) for ethanol is more than 100 times that of catalyst Pd@RGO. The mass activity values of the two catalysts in 0.5 M NaOH with 1 M ethanol solution are 376.71 and 3.59 mA mgPd^−1^, respectively. For the prepared platinum-based catalyst, in the methanol electrocatalytic reaction in an acidic electrolyte, the catalytic activity of Pt@RGO (with H_2_) is much higher than that of Pt@RGO, similar to the trend of palladium-based catalyst supported on reduced graphene oxide. The surface of tiny nanoparticles synthesized without hydrogen gas is surrounded by CTAB molecules and adsorbed by graphene oxide. Therefore, the active sites of the catalyst are covered, while the catalyst for hydrogen reduction is very different.

**Figure 5:**
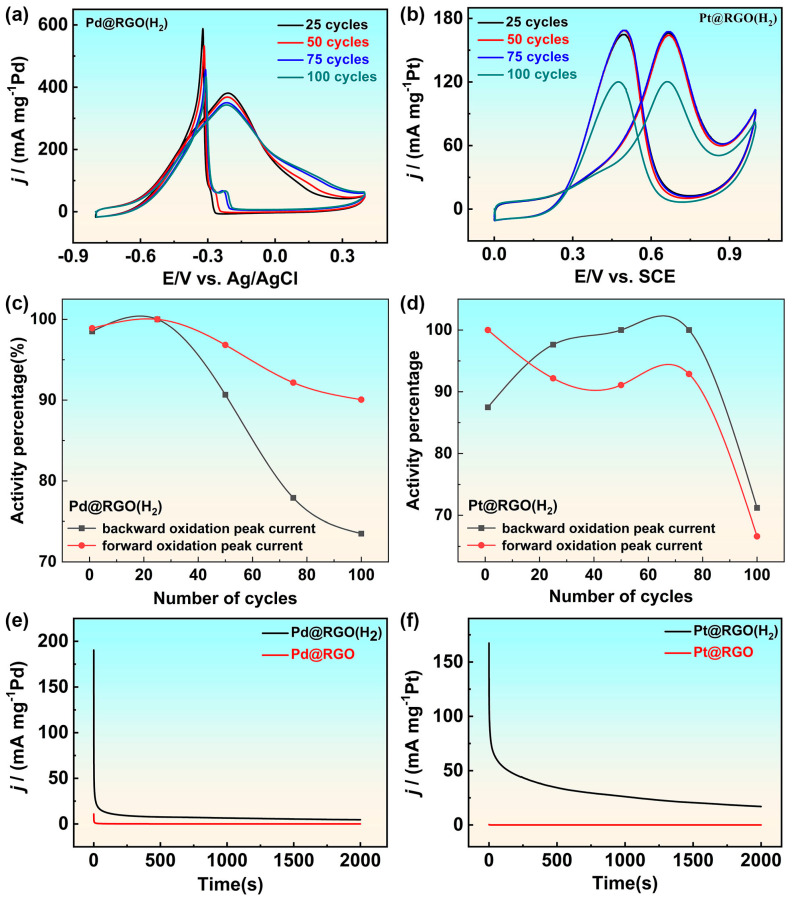
Repetitive CV curves of (**a**) Pd@RGO (with H_2_) in 1 M NaOH with 1 M ethanol solution, (**b**) Pt@RGO (with H_2_) in 0.5 M H_2_SO_4_ with 0.5 M methanol solution, (**c**) The percentage change of peak current in forward and backward scanning, during 100 repeated cyclic voltammetry tests of Pd@RGO (with H_2_) in alkaline electrolyte, (**d**) The percentage change of peak current in forward and backward scanning, during 100 repeated cyclic voltammetry tests of Pt@RGO (with H_2_) in acidic electrolyte, (**e**) Chronoameperometric (CA) activity of Pd@RGO (with H_2_) and Pd@RGO in 1 M NaOH with 1 M ethanol solution, (**f**) CA responses of Pt@RGO (with H_2_) and Pt@RGO in 0.5 M H_2_SO_4_ with 0.5 M methanol solution.

Stability is a crucial indicator for catalysts. Repetitive cyclic voltammetry and chronoamperometry tests were used to evaluate the stability of catalysts. As seen in Figure 5a,c, in the repeated cyclic voltammetry test of 100 cycles, the electrochemical activity of the palladium-based catalyst slowly decreased, but Pd@RGO (with H_2_) still maintained 90% of its original activity after the test. However, the situation was different for platinum-based catalysts. After repeated cyclic voltammetry testing, the platinum-based catalyst loaded with reduced graphene oxide lost one-third of its initial activity, Compared with catalysts Pd@RGO and Pt@RGO, the 2000s chronoamperometry test also proves that the graphene-loaded nanoflowers catalyst prepared has good stability.

## 3. Experimental Section

### 3.1. Chemicals and Materials

Anhydrous ethanol, sodium chloride, sulfuric acid, sodium hydroxide, potassium chloroplatinate, anhydrous ethanol, and CTAB were purchased from Sinopharm Chemical Reagent Co., Ltd. (Shanghai, China). Palladium chloride. The (PdCl_2_, 59.5%) was purchased from Shanghai Zhiyu New Material Co., Ltd. (Shanghai, China). Graphene oxide (GO) was obtained from Suzhou Global Technology Co., Ltd. (Suzhou, China). Potassium hexachloroplatinate (K_2_PtCl_6_, 98%) was purchased from Shanghai Macklin Biochemical Technology Co., Ltd. (Shanghai, China). High-purity hydrogen and high-purity nitrogen were purchased from Guangzhou Junduo Gas Co., Ltd. (Guangzhou, China). All reagents were analytical grade and used without further purification.

### 3.2. Preparation of Palladium and Platinum-Based Catalysts Supported on Reduced Graphene Oxide

CTAB solution (44 mg CTAB and 2 mL H_2_O), NaOH solution (13 mg NaOH mixed with 1 mL H_2_O), and Na_2_PdCl_4_ solution (9 mg PdCl_2_ and 6 mg NaCl added to 2 mL H_2_O) were sequentially added to GO dispersion (22 mg GO and 32 mL H_2_O), and the mixture was stirred for 30 min, 10 min, and 10 min, respectively. After hydrogen gas was introduced into the mixture for 20 min, the temperature was slowly raised to 90 °C and maintained for 2 h, and the solution gradually turned black. After the temperature of the mixed solution descended to room temperature, ethanol, and deionized water were used for ultrasonic washing of the centrifugal separation catalyst. The upper clear liquid was colorless and transparent, while the lower product was a black solid. The obtained material was repeatedly washed at least five times for analysis, characterization, and electrochemical testing, and the sample was labeled as Pd@RGO (with H_2_).

The preparation process without introducing H_2_ was the same as above. After centrifugation, the upper liquid appeared light yellow, and the lower product was a yellow-brown solid. The obtained material was washed four to five times repeatedly for analysis, characterization, and electrochemical testing. The sample was labeled as Pd@RGO.

The synthesis and cleaning procedure of platinum-based catalyst loaded on reduced graphene oxide was the same as the above process, and the sample was labeled as Pt@RGO and Pt@RGO (with H_2_). The different feeding amounts are as follows: CTAB solution (44 mg CTAB and 6 mL H_2_O), NaOH solution (13 mg NaOH mixed with 1 mL H_2_O), and K_2_PtCl_6_ solution (24.29 mg and 2 mL H_2_O) were sequentially added to GO dispersion (39 mg GO and 60 mL H_2_O).

### 3.3. Electrochemical Performance of Prepared Platinum and Palladium-Based Catalyst

A 3 mm diameter glassy carbon electrode was selected as the working electrode and the surface was polished with 0.05 µm α-Al_2_O_3_. Then, ultrapure water was used to rinse the electrode surface, and after drying at room temperature, a glassy carbon electrode with a mirror surface was obtained. After ultrasonic dispersion, 5 µL dispersion (2.5 mg mL^−1^) of catalyst suspension and 1 µL Nafion solution (5%) were transferred and dropped onto the surface of the treated glassy carbon electrode, respectively. Graphene-supported platinum or palladium-based catalyst-modified glassy carbon electrode was obtained after natural drying.

A platinum foil electrode (1 cm × 1 cm) was used as an auxiliary electrode, and an Ag/AgCl electrode (alkaline environment) or calomel electrode (acidic environment) was used as a reference electrode. The CHI660e electrochemical workstation was used for electrocatalytic experiments, conducting cyclic voltammetry (CV), current versus time (i-t), and 100 cycle CV tests in 0.5 M NaOH solution and 0.5 M NaOH + 1 M ethanol solution, respectively. The electrochemical testing under acidic conditions includes CV scanning, i-t testing, and 100-cycle CV testing using 0.5 M H_2_SO_4_ solution and 0.5 M H_2_SO_4_ + 0.5 M methanol solution. Before the test, high-purity N_2_ was introduced into the electrolyte for 15 min to remove oxygen, and the test was conducted at room temperature.

### 3.4. Characterization

The morphology of graphene-supported palladium and platinum-based catalysts obtained was observed using a transmission electron microscope (TEM, Tecnai F20, FEI, Eindhoven, The Netherlands) with an acceleration voltage of 120 kV. The crystal structure of the catalysts was characterized using an X-ray powder diffractometer (XRD, D/max 2500PC, perkinelmer, Austin, TX, USA) with a copper target, a scanning range between 5° and 90°, and a scanning speed of 5°/min. The Raman spectra of GO, Pt@RGO, Pd@RGO, Pd@RGO (with H_2_), and Pt@RGO (with H_2_) were obtained using a confocal Raman spectrometer (RM2000, Renishaw, New Mills, UK). The composition of reduced graphene-supported platinum and palladium-based catalysts was analyzed by an inductively coupled plasma-mass spectroscopy (ICP-MS, Varian 710-ES, Varian, Atlanta, GA, USA).

## 4. Conclusions

In summary, a synthesis procedure for graphene-supported flower-shaped platinum and palladium-based nanocatalysts was developed, with CTAB as a stabilizer, active hydrogen atom as a reducing agent, and graphene oxide as the carrier. The metal nanostructures reduced by hydrogen serve as catalysts for the decomposition of hydrogen into active hydrogen atoms. The initial one-dimensional nanoparticles grow in an anisotropic direction through adhesion and ultimately assemble into a three-dimensional mesoporous catalyst under the presence of strong reducing agents. The abundant pores in the catalyst provide more flow channels for reactants and products to contact active sites, exhibiting enhanced electrocatalytic activity for methanol and ethanol in both acidic and alkaline electrolytes, while rapidly improving stability.

## Data Availability

Data are contained within the article.

## References

[1] Sazali N., Salleh W.N.W., Jamaludin A.S., Razali M.N.M. (2020). New perspectives on fuel cell technology: A brief review. Membranes.

[2] Mekhilef S., Saidur R., Safari A. (2012). Comparative study of different fuel cell technologies. Renew. Sustain. Energy Rev..

[3] Han M., Kani K., Na J., Kim J., Bando Y., Ahamad T., Alshehri S.M., Yamauchi Y. (2023). Retrospect and prospect: Nanoarchitectonics of platinum-group-metal-based materials. Adv. Funct. Mater..

[4] Zhao X., Sasaki K. (2022). Advanced Pt-based core–shell electrocatalysts for fuel cell cathodes. Acc. Chem. Res..

[5] Sajid A., Pervaiz E., Ali H., Noor T., Baig M.M. (2022). A perspective on development of fuel cell materials: Electrodes and electrolyte. Int. J. Energy Res..

[6] Zhang Y., Gao F., You H., Li Z., Zou B., Du Y. (2022). Recent advances in one-dimensional noble-metal-based catalysts with multiple structures for efficient fuel-cell electrocatalysis. Coord. Chem. Rev..

[7] Xu Q., Niu Y., Li J., Yang Z., Gao J., Ding L., Ni H., Zhu P., Liu Y., Tang Y. (2022). Recent progress of quantum dots for energy storage applications. Carb. Neutrality.

[8] Farrag M., Ali G.A.M. (2022). Hydrogen generation of single alloy Pd/Pt quantum dots over Co_3_O_4_ nanoparticles via the hydrolysis of sodium borohydride at room temperature. Sci. Rep..

[9] Ye M., Gong J., Lai Y., Lin C., Lin Z. (2012). High-efficiency photoelectrocatalytic hydrogen generation enabled by palladium quantum dots-sensitized TiO_2_ nanotube arrays. J. Am. Chem. Soc..

[10] Doan H., Morais T., Borchtchoukova N., Wijsboom Y., Sharabi R., Chatenet M., Finkelshtain G. (2022). Bimetallic Pt or Pd-based carbon supported nanoparticles are more stable than their monometallic counterparts for application in membraneless alkaline fuel cell anodes. Appl. Catal. B.

[11] Huang S., Lu S., Hu H., Xu F., Li H., Duan F., Zhu H., Gu H., Du M. (2021). Hyper-dendritic PdZn nanocrystals as highly stable and efficient bifunctional electrocatalysts towards oxygen reduction and ethanol oxidation. Chem. Eng. J..

[12] Liu C., Cai X., Wang J., Liu J., Riese A., Chen Z., Sun X., Wang S.-D. (2016). One-step synthesis of AuPd alloy nanoparticles on graphene as a stable catalyst for ethanol electro-oxidation. Int. J. Hydrogen Energy.

[13] Ji L., Zhang X., Qian N., Li J., Shen S., Wu X., Tan X., Zhang H., Yang D. (2024). A universal synthesis strategy of Pd-based trimetallic nanowires for efficient alcohol electrooxidation. Nanoscale.

[14] Xie R., Zhou L., Chen D., Yan S., Su N., Zhang Y. (2024). Facile construction of core-shell AuPd nanochain networks in one-pot for ethanol oxidation. Int. J. Hydrogen Energy.

[15] Li S., Jin H., Wang Y. (2023). Recent progress on the synthesis of metal alloy nanowires as electrocatalysts. Nanoscale.

[16] Wang Q., Liu J., Li T., Zhang T., Arbiol J., Yan S., Wang Y., Li H., Cabot A. (2022). Pd_2_Ga nanorods as highly active bifunctional catalysts for electrosynthesis of acetic acid coupled with hydrogen production. Chem. Eng. J..

[17] Zhao Y., Sarhan R.M., Eljarrat A., Kochovski Z., Koch C., Schmidt B., Koopman W., Lu Y. (2022). Surface-Functionalized Au–Pd nanorods with enhanced photothermal conversion and catalytic performance. ACS Appl. Mater. Interfaces.

[18] Sun Y.-B., Ni M., Chi C., Yang D.-R., Chen X.-L., Qi Q., Li J., Xia X.-H. (2023). Plasmon driven super-high HER activity of electronic structure and lattice strain engineered single atomic layer Pd@ Au nanorods. Chem. Eng. J..

[19] Shi L., Wang Q., Ren Q., Yang Q., Zhao D., Feng Y., Chen H., Wang Y. (2022). Facile synthesis of Pd and PdPtNi trimetallic nanosheets as enhanced oxygen reduction electrocatalysts. Small.

[20] Ando S., Yamamoto E., Kobayashi M., Kumatani A., Osada M. (2024). Facile Synthesis of Pd Nanosheets and Implications for Superior Catalytic Activity. ACS Nano.

[21] Huang X., Xu B., Feng J., Hu S., Dou W., Yang T., Zhan C., Liu S., Ji Y., Li Y. (2023). Continuous Phase Regulation of a Pd–Te Hexagonal Nanoplate Library. J. Am. Chem. Soc..

[22] Yadav V., Jeong S., Ye X., Li C.W. (2022). Surface-limited galvanic replacement reactions of Pd, Pt, and Au onto Ag core nanoparticles through redox potential tuning. Chem. Mater..

[23] Li Z., Xie Z., Zhang Y., Mu X., Xie J., Yin H.-J., Zhang Y.-W., Ophus C., Zhou J. (2023). Probing the atomically diffuse interfaces in Pd@ Pt core-shell nanoparticles in three dimensions. Nat. Commun..

[24] Ou Z., An Z., Ma Z., Li N., Han Y., Yang G., Jiang Q., Chen Q., Chu W., Wang S. (2023). 3D Porous graphene-like carbons encaged single-atom-based Pt for ultralow loading and high-performance fuel cells. ACS Catal..

[25] Gong Y., Lv F., Lu Y., Yu Y., Niu J., Lang J., Deng Y., Cao X., Gu H. (2020). In Situ surface-derivation of AgPdMo/MoS_2_ nanowires for synergistic hydrogen evolution catalysis in alkaline solution. Nanoscale.

[26] Hakkeem H.M.A., Babu A., Shilpa N., Venugopal A.A., Mohamed A.P., Kurungot S., Pillai S. (2022). Tailored synthesis of ultra-stable Au@ Pd nanoflowers with enhanced catalytic properties using cellulose nanocrystals. Carbohyd. Polym..

[27] Xie R., Lu S., Deng Y., Mei S., Cao X., Zhou L., Lan C., Gu H. (2019). Facile synthesis of sea-urchin-like Pt and Pt/Au nanodendrites and their enhanced electrocatalytic properties. Inorg. Chem..

[28] Lee S.J., Jang H., Lee D.N. (2023). Recent advances in nanoflowers: Compositional and structural diversification for potential applications. Nanoscale Adv..

[29] Xie R., Qin F., Zhou L., Chen D., Liu J., Shen J., Yan S. (2024). Supported palladium nanocubes reduced by graphene oxide and surface-cleaned for enhanced electrocatalytic activity. J. Alloys Compd..

[30] Gautam S., Hadley A.M.K., Gates B.D. (2022). Controlled Growth of Platinum Nanoparticles during Electrodeposition Using Halide Ion Containing Additives. J. Electrochem. Soc..

[31] Qiu P., Lian S., Yang G., Yang S. (2017). Halide ion-induced formation of single crystalline mesoporous PtPd bimetallic nanoparticles with hollow interiors for electrochemical methanol and ethanol oxidation reaction. Nano Res..

[32] Wang D., Zhang Y., Zhang K., Wang X., Wang C., Li Z., Gao F., Du Y. (2023). Rapid synthesis of Palladium-Platinum-Nickel ultrathin porous nanosheets with high catalytic performance for alcohol electrooxidation. J. Colloid Interface Sci..

[33] Xing Q., Yan W., Wei C., Xiao Z., Jin Z., Liu X., Ren J., Chen Y., Li X. (2022). Controlled Preparation and Anti-Sulfate Electrocatalysis of Self-Assembled Multidimensional PtZn Quasi-Cubic Nanodendrites. Adv. Mater. Interfaces.

[34] Zi X., Wang R., Liu L., Dai H., Zhang G., He H. (2011). Cetyltrimethylammonium bromide assisted preparation and characterization of pd nanoparticles with spherical, worm-like, and network-like morphologies. Chin. J. Catal.

[35] Gogoi R., Nath R., Borah G. (2024). CTAB-assisted synthesis of reduced graphene oxide supported Pd nanoparticles (Pd@ rGO) as a sustainable heterogeneous catalyst for C-2 arylation of indoles with arylboronic acids. J. Organomet. Chem..

[36] Shen L., Tu F., Shang Z., Ma M., Xia Y., Zhao Z., Zhao L., Wang Z., Shao G. (2022). Surfactant-assisted synthesis of platinum nanoparticle catalysts for proton exchange membrane fuel cells. Int. J. Hydrogen Energy.

[37] Xie R., Pan Y., Gu H. (2015). Synthesis of Pt dendritic nanocubes with enhanced catalytic properties. RSC Adv..

[38] Chang L., Cao Y., Peng W., Li C., Fan G., Song X., Shi X. (2020). Efficiently removing cetyl trimethyl ammonium bromide from wastewater by graphene oxide. Surf. Interface Anal..

[39] Konda S.K., Chen A. (2016). Palladium based nanomaterials for enhanced hydrogen spillover and storage. Mater. Today.

[40] Pham V.H., Dang T.T., Singh K., Hur S.H., Shin E.W., Kim J.S., Lee M.A., Baeck S.H., Chung J.S. (2013). A catalytic and efficient route for reduction of graphene oxide by hydrogen spillover. J. Mater. Chem..

[41] Alkhouzaam A., Abdelrazeq H., Khraisheh M., AlMomani F., Hameed B.H., Hassan M.K., Al-Ghouti M.A., Selvaraj R. (2022). Spectral and structural properties of high-quality reduced graphene oxide produced via a simple approach using tetraethylenepentamine. Nanomaterials.

[42] Jin H., Zhu L., Jin S., Jiang L., Zou Y. (2021). Raman spectroscopy analysis of graphene oxide-enhanced textiles. J. Raman Spectrosc..

[43] De Silva K.K.H., Viswanath P., Rao V.K., Suzuki S., Yoshimura M. (2021). New insight into the characterization of graphene oxide and reduced graphene oxide monolayer flakes on Si-based substrates by optical microscopy and raman spectroscopy. J. Phys. Chem. C.

[44] Lee A.Y., Yang K., Anh N.D., Park C., Lee S.M., Lee T.G., Jeong M.S. (2021). Raman study of D* band in graphene oxide and its correlation with reduction. Appl. Surf. Sci..

[45] Kaniyoor A., Ramaprabhu S. (2012). A Raman spectroscopic investigation of graphite oxide derived graphene. AIP Adv..

[46] Minitha C.R., Rajendrakumar R.T. (2013). Synthesis and characterization of reduced graphene oxide. Adv. Mater. Res..

[47] Khan M.F., Cazzato G., Saleemi H.A., Macadangdang R.R., Aftab M.N., Ismail M., Khalid H., Ali S., Bakhtiar S.U.H., Ismail A. (2022). Sonophotocatalytic degradation of organic pollutant under visible light over Pt decorated CeO_2_: Role of ultrasonic waves for unprecedented degradation. J. Mol. Struct..

[48] Yang J., Shi C., Liu F., Zhu E., Wei D., Ren Y., Chen R., Xu M. (2024). High-index facets Pt concave nanocubes with small interface angles induced by N-defective sites in the integrated electrode for methanol oxidation. J. Power Sources.

[49] Qiao M., Wei Y., Dong Y.-J., Wang J.-X., Chen J.-F. (2024). A Universal Approach for Controllable Synthesis of Homogeneously Alloyed PtM Nanoflowers toward Enhanced Methanol Oxidation. Small.

[50] Baran N.Y., Baran T., Çalışkan M. (2021). Production of Pd nanoparticles embedded on micro-sized chitosan/graphitic carbon nitride hybrid spheres for treatment of environmental pollutants in aqueous medium. Ceram. Int..

[51] Makin A.M., Nsengiyumva W., Bizuneh G.G., Xu Z., Zhang G. (2023). Stabilizing agents assisted construction of monometallic self-supporting Palladium NCs with ultrafine nanostructures and rich surface area for highly efficient direct ethanol fuel cell. J. Electroanal. Chem..

[52] Kumar G., Das S.K., Nayak C., Dey R.S. (2024). Pd “kills two birds with one stone” for the synthesis of catalyst: Dual active sites of Pd triggers the kinetics of O_2_ electrocatalysis. Small.

[53] Geng Z.-G., Zhang G.-H., Lin Y., Yu X.-X., Ren W.-Z., Wu Y.-K., Pan N., Wang X.-P. (2012). A green and mild approach of synthesis of highly-conductive graphene film by zn reduction of exfoliated graphite oxide. J. Chem. Phys..

[54] Galvão F.M.F., Cabral R.L.B., Santos E.V., Santos J.E.L., Santos T.F., Zille A., Mattos A.L.A., Souza D.F.S., Nascimento J.H.O. (2023). Comparative Study of the Synthesis and Characterization of Reduced Graphene Oxide (RGO) Using an Eco-Friendly Reducing Agent. J. Electron. Mater..

[55] Huang H.-H., De Silva K.K.H., Kumara G.R.A., Yoshimura M. (2018). Structural evolution of hydrothermally derived reduced graphene oxide. Sci. Rep..

